# From Promise to Precision: Future Directions for Smartphone Application Eating Disorder Interventions—Commentary on Cruz et al. (2025)

**DOI:** 10.1002/eat.24537

**Published:** 2025-08-29

**Authors:** Mariel Messer

**Affiliations:** ^1^ School of Psychology Deakin University Burwood Australia

**Keywords:** digital mental health, digital phenotyping, eating disorders, hybrid care, machine learning, mobile health (mHealth), smartphone applications, stepped‐care

## Abstract

Despite growing interest in smartphone applications (apps) as tools for eating disorder (ED) intervention, significant challenges remain in optimizing their utility and implementation. Cruz et al. conducted a meta‐analysis of 14 trials examining smartphone app‐based interventions for EDs, finding apps to effectively reduce numerous symptoms. While their findings highlight the promise of app‐based interventions for symptom management, realizing their full potential will require a shift toward enhancing their potency, personalization, and flexible delivery. This commentary proposes three critical areas for future research in apps within the context of ED care. First, there is a pressing need to transition from static to adaptive systems that tailor support based on real‐time, continuously collected active, passive, and metadata. Second, greater consideration should be given to how apps are best deployed, whether through established or novel models of care. Third, outcome variability points to a need for precision‐focused research to better identify who benefits most from app‐based interventions, ideally leveraging complex data‐driven approaches that model clinical, behavioral, and engagement data. Together, these directions chart a pathway toward more intelligent, responsive, and equitable app‐based tools for EDs.

1

**Summary**

- This commentary proposes future directions for smartphone apps in eating disorder care, building on a recent meta‐analysis showing they can reduce symptoms.
- Adaptive, AI‐powered systems and integration into stepped or hybrid care could enhance effectiveness and reach.
- Prediction models using multimodal data may help identify who benefits most, and safeguard against harm.
- Rigorous testing and equitable implementation are essential to realize their full potential.

## 2 Introduction

Individuals with eating disorders (EDs) face barriers to care, such as time constraints, difficulty recognizing symptoms, and lack of personalized support, leading to poor recovery rates. Smartphone applications (apps) offer a promising solution, providing flexible, scalable, and real‐time intervention. Interest in app‐based interventions for EDs has surged, with trials evaluating their effectiveness in clinical and subclinical populations. A 2025 meta‐analysis by Cruz et al., covering 14 randomized trials, found that these apps significantly reduced several symptoms in subclinical groups. In clinical populations, only objective binge eating showed significant effects when apps were part of guided self‐help programs. While apps show promise for ED management, they are not universally effective, often lack personalization, and are rarely integrated into standard treatments (with the exception of the Recovery Record app). Future research must focus on optimizing app design and delivery to better support individuals with EDs. Therefore, to contribute to this evolving conversation, the purpose of this commentary is to identify three key priority areas for future research in this context.

## 3 Future Directions

### 3.1 Moving From Static to Adaptive Apps That Integrate AI‐Powered Functionality

Most ED apps are static, one‐size‐fits‐all designs, offering limited capacity for a high degree of personalization. Users are typically guided through the same content regardless of their symptom profile, treatment history, or momentary needs. This uniform approach may help explain some of the outcome variability found (Cruz et al. 2025). Emerging evidence suggests that tailoring intervention content to match users' evolving needs could enhance treatment engagement and potency.

Modern smartphones contain advanced AI technologies that can support adaptive systems responsive to users in real time. The next step is embedding large language models (LLMs) so these tools function as fully adaptive systems. LLMs can process multimodal data to build a richer, real‐time picture of users and dynamically adjust intervention content, tone, and timing. Beyond app integration, they can triage clients, aid diagnosis, and predict or respond to deterioration or improvement when embedded in clinician dashboards, telehealth systems, or service websites. The goal is not to create more apps in an already saturated marketplace, but to refine evidence‐based ED apps by incorporating these AI capabilities. Moving toward personalized, data‐driven tools, whether apps with embedded LLMs or other AI‐powered systems, represents a critical step in enhancing the effectiveness, relevance, and scalability of digital interventions for EDs.

To build intelligent and adaptive apps, it is necessary to first develop data‐driven algorithms that can predict when an individual is approaching a period of heightened risk. Early detection is essential for enabling just‐in‐time interventions that are preventive rather than reactive. While some existing ED apps have made initial attempts to incorporate prediction‐based features (e.g., Recovery Record), these have largely relied on periodic self‐report symptom scales. Self‐report measures are limited by recall bias and fluctuations in user insight, making them suboptimal for detecting the subtle and dynamic changes that often precede clinical deterioration.

To improve predictive precision, future efforts should integrate multiple data streams supported by smartphone technology. Data streams can be active or passive in nature. Active data includes clinical rating scales, symptom trackers, and diary entries, all of which are directly input by the user. These data sources have the advantage of capturing ecologically valid and momentarily relevant indicators of risk. Passive data involves continuous and unobtrusive collection of behavioral and environmental information through in‐built smartphone sensors such as GPS, accelerometers, and usage patterns (Linardon and Torous 2025). Although many ED risk behaviors occur in private, GPS data could reveal frequent visits to gyms or diet clinics, avoidance of food‐related settings, reduced movement variability suggesting social withdrawal, or sudden changes in routines. Combined with self‐report and other passive signals, these indicators could help detect emerging risk and enable timely, context‐appropriate interventions. Passive data can uncover nuanced behavioral shifts that are not captured by self‐report and may provide early warning signs of symptom relapse or mental health decline. By combining both active and passive data sources, predictive models may be more sensitive and accurate, forming the foundation for highly personalized and timely interventions.

Modeling both passive and active data inputs may enable the construction of highly individualized digital phenotypes capable of detecting early warning signs of clinical deterioration in EDs. An intelligent smartphone system with embedded LLM capabilities or other AI‐powered software could integrate these data streams to tailor its response to the user's unique behavioral and symptom profile. For example, a user reporting increased weight and shape preoccupation (via active inputs) with reduced physical activity and social interactions (from passive signal anomalies) might receive prompts for valued activities or body‐neutral practices. Conversely, a user with the same preoccupation but increased screen time on diet‐related social media might be offered a thought‐restructuring exercise targeting social comparisons.

This level of personalization is now technically feasible, but before this can become a clinical reality, effort should focus on constructing these multi‐data prediction models so that they can serve as tailoring variables within just‐in‐time interventions. Achieving this will be essential for realizing the full potential of smartphone technology to deliver more precise, responsive, and effective care for individuals with EDs.

### 3.2 Consider Current and Emerging Care Models for App Integration

The optimal way to integrate smartphone apps into ED care remains unclear. Apps can serve as standalone tools for symptom management, particularly for those without access to specialized support, with growing evidence for self‐guided approaches. Alternatively, apps like Recovery Record, when embedded in routine care, enhance self‐monitoring, patient‐clinician communication, and treatment engagement alongside traditional therapy. Apps also support between‐session “homework” practice. However, current efforts have only scratched the surface of their potential. Innovations like digital navigators to boost uptake, multimodal data integration for just‐in‐time prompting, or artificial intelligence‐driven predictive analytics to anticipate symptom escalation could transform care delivery. These advancements pave the way for exploring innovative care models, such as stepped care and hybrid care, to broaden access and improve outcomes for individuals with EDs.

In the stepped care approach, interventions are sequenced according to patient needs and response, where lower intensity interventions are typically offered first, with higher level care offered next if a person does not derive benefit from the initial intervention. In this framework, a self‐guided app could be deployed as an initial, low‐intensity intervention for individuals with emerging symptoms or low clinical severity. Those who do not experience meaningful improvement could then be “stepped up” to higher‐intensity services, such as outpatient specialist treatment. This could improve treatment efficiency by reducing unnecessary clinician time for individuals who may benefit from lower‐level intervention.

Despite its theoretical appeal, stepped‐care models that incorporate apps as a first step have not been empirically tested in the ED field, so their value and clinical utility remain an open question. Thus, well‐powered adaptive trials that start individuals off with an app, monitor progress, and then re‐randomize to higher level care if needed are important future directions to see how apps could be situated within existing treatment models.

In the hybrid care model, apps are embedded within the clinical workflow to complement, rather than replace, traditional services (Macrynikola et al. 2025). Unlike earlier uses of digital health apps that primarily aim to support between‐session homework compliance, hybrid models position apps as central to care delivery by enabling highly personalized treatment plans that respond to both patient and clinician needs. These apps facilitate real‐time monitoring and intervention between sessions by collecting active self‐reports and passive sensor data to detect subtle shifts in behavior or symptom risk. Importantly, these data are fed directly into clinician dashboards, which generate automated alerts when atypical patterns or signs of deterioration emerge. This allows clinicians to promptly intervene by following up with the patient, adjusting the care plan, or recommending additional resources.

Preliminary evidence supports the feasibility, acceptability, and augmented efficacy of hybrid care models for emotional disorders (Macrynikola et al. 2025). Extending this approach to ED care could enable earlier intervention, enhance personalization, and reduce burden for both clients and clinicians. While hybrid care models may be especially useful for ED treatment, it is important to acknowledge that a lot of foundational and pilot work is needed (e.g., usability testing of clinical dashboards, development of accurate predictive algorithms to inform risk status, and clinician barriers to dissemination) to ensure such models can become a clinical reality. Importantly, stepped and hybrid care models requiring clinician involvement may not be feasible for underserved populations. Alternative integration points, such as primary care providers, emergency departments, community health centers, or telehealth and mental health hotlines, could facilitate delivery of app‐based support for individuals in rural or low‐resource settings. It is hoped that this commentary fosters discussion and collaboration to help expedite this idea.

### 3.3 Move Toward Precision Digital Care: Predicting Who Benefits From ED Apps

Apps can be effective tools to manage symptoms, but it is crucial to acknowledge that not all individuals will benefit equally. Digital formats may be a lifeline for some, but for others, they may fall short of providing the depth, support, or personalization required. Importantly, a small subset of individuals may even experience adverse effects, such as increased preoccupation with symptoms, heightened distress, or clinical deterioration. As these tools become more widely implemented, identifying the characteristics, such as age, gender identity, cultural background, and smartphone literacy, that influence engagement and outcomes will be essential for ensuring interventions are appropriately matched to individual needs.

To date, attempts to identify predictors of treatment response to digital health apps have largely been unsuccessful (McClure et al. 2024). This is because prior efforts have focused on a very small number of potential predictors in isolation, which produce very small explanatory power. One way to overcome this prior piecemeal approach is to build complex, data‐driven machine learning models that explore the combined predictive power (linear and non‐linear) of many variables at once. These variables could include active self‐report data from rating scales, passive behavioral signals from smartphone sensors, and language‐based data streams processed through large LLMs. When embedded within an app, such systems could integrate these multimodal inputs to construct a richer, real‐time profile of the user's current state and likelihood of treatment response. Unlike traditional regression models that typically assess variables one at a time, machine learning can identify complex, non‐linear relationships across multiple features, enabling the development of individual‐level clinical prediction models (McClure et al. 2025). Importantly, any predictive modeling whether—via LLMs or other AI approaches should—be tested alongside evidence‐based treatments to ensure clinical relevance and safety. However, if successful, these models could forecast, with greater precision, who is likely to respond positively to app‐based interventions, who might require a different level or type of care, and who may be at risk of harm.

Indeed, evidence from other mental health fields shows that machine learning techniques that model multimodal data sources can predict patient response to digital interventions with up to 90% accuracy. While initial attempts have been made to explore the feasibility of using machine learning to predict outcomes from ED apps, this work is largely exploratory and proof of concept; confirmatory studies that prospectively validate promising algorithms in new samples are needed to fully understand the types of people likely to respond (or not) to apps. Advancing this line of research will be essential for enabling precision digital ED care, where app‐based interventions can be proactively matched to the individuals most likely to benefit.

## 4 Conclusion

The meta‐analysis by Cruz et al. highlights the potential of smartphone apps in ED care but reveals that their capabilities are only beginning to be explored. To become intelligent, scalable systems that adapt to users' needs in real time, apps require significant innovation, integration, and rigorous testing. This commentary provides key insights to advance this vision.

## Author Contributions

**Mariel Messer:** conceptualization, writing – original draft, writing – review and editing.

## Ethics Statement

The author has nothing to report.

## Conflicts of Interest

The author declares no conflicts of interest.

## Data Availability

Data sharing not applicable to this article as no datasets were generated or analysed during the current study.
